# Positron Emission Tomography imaging of tumor necrosis factor in lung injury

**DOI:** 10.48101/ujms.v131.13817

**Published:** 2026-04-28

**Authors:** Olivia Wegrzyniak, Huayi Han, Olof Eriksson

**Affiliations:** Science for Life Laboratory, Department of Medicinal Chemistry, Uppsala University, Uppsala, Sweden

**Keywords:** Tumor necrosis factor, inflammatory diseases, positron emitted tomography, molecular imaging, inflammation

## Abstract

**Introduction:**

Tumor necrosis factor (TNF) is a key pro-inflammatory cytokine involved in various pulmonary diseases, including idiopathic pulmonary fibrosis (IPF), where it contributes to immune cell recruitment, tissue remodeling, and disease progression. Despite the therapeutic potential of TNF-targeting strategies, the lack of non-invasive tools to assess TNF activity in the lungs limits personalized treatment and trial stratification. This study aimed to evaluate the novel Affibody molecule-based positron emission tomography (PET) tracer [^68^Ga]Z_0185_, targeting TNF, for its ability to detect inflammation in vivo using the bleomycin (BLM)-induced lung injury model in rats.

**Methods:**

DOTA-Z_0185_ was generated by solid phase peptide synthesis, and a method for labeling by Gallium-68 was developed. The resulting PET tracer [^68^Ga]Z_0185_ was evaluated for binding to recombinant TNF by a radioimmuno-assay. [^68^Ga]Z_0185_ was further evaluated by PET imaging and ex vivo biodistribution studies in a bleomycin rat model of lung injury in comparison with healthy rats.

**Results:**

DOTA-Z_0185_ was consistently radiolabeled with a radiochemical purity of at least 95%. [^68^Ga]Z_0185_ bound to recombinant human TNF in vitro with a mechanism that could be partially inhibited by etanercept (131.8 ± 12.0 vs. 74.2 ± 5.6 fmol, *P* < 0.05). [^68^Ga]Z_0185_ uptake was significantly higher in injured pulmonary regions in BLM-treated rats compared to lung tissue in control animals (SUV_mean_ 0.58 ± 0.22 vs. 0.25 ± 0.07, *P* < 0.05) as analyzed by PET/computed tomography (CT) in vivo imaging. These regions corresponded with histologically confirmed areas of inflammation, with dense CD68+ macrophage infiltration.

**Conclusion:**

[^68^Ga]Z_0185_ enables non-invasive detection of localized TNF-driven inflammation in the lung. This approach offers a promising imaging tool for patient stratification, therapy monitoring, and guiding anti-TNF interventions in pulmonary diseases.

## Background

Tumor necrosis factor (TNF) is a proinflammatory cytokine primarily produced by monocytes and macrophages. It plays a central role in various inflammatory diseases, including rheumatoid arthritis, inflammatory bowel disease, as well as in pulmonary conditions (1) such as idiopathic pulmonary fibrosis (IPF) and sarcoidosis. To date, four TNF-blocking biologic drugs (infliximab, adalimumab, certolizumab pegol, and etanercept) have been approved for clinical use.

Despite the well-established involvement of TNF in pulmonary diseases and its elevated expression in affected lung tissue, clinical responses to anti-TNF therapies have been inconsistent (2–5) This is likely due to the heterogeneity of underlying disease mechanisms and the lack of diagnostic tools to identify patients with TNF-driven inflammation.

Currently, [^18^F]Fludeoxyglucose (FDG) positron emission tomography (PET) is a standard imaging technique used to assess pulmonary inflammation (6). However, as it reflects general glucose metabolism, it lacks the specificity to distinguish TNF-mediated inflammation from other immune responses. This limits its utility in guiding patient selection or monitoring therapeutic efficacy of TNF-targeted treatments.

Therefore, we investigated a novel PET imaging approach using [^68^Ga]Z_0185_, based on an Affibody molecule (Z_0185_) specifically engineered to bind human recombinant TNF with subnanomolar affinity (K_D_ = 0.1–0.5 nM for human TNF) (7). Due to their small size and rapid tissue penetration, Affibody molecules offer favorable pharmacokinetics for PET imaging. DOTA-conjugated Z_0185_ retains nanomolar affinity to TNF (K_D_ = 1.1 nM) and is in preclinical evaluation as a PET imaging diagnostic for use in rheumatoid arthritis (8). Z_0185_ was selected by phage display toward the single chain TNF. The exact binding epitope on TNF is not known, but overlaps with Etanercept and the TNF receptor since they compete for binding (7). Furthermore, dimerization or trimerization of Z_0185_ can dramatically increase affinity (7), indicating that avidity effects may enable improved binding toward the bioactive TNF trimer.

In this study, we assessed the ability of [^68^Ga]Z_0185_ to non-invasively detect TNF expression in a rat model of bleomycin (BLM)-induced lung injury, with the aim of validating its potential as a diagnostic tool for TNF-driven pulmonary inflammation.

## Methods

### Chemical synthesis of DOTA-Z_0185_

The 58-amino acid peptide Z_0185_ Affibody molecule was produced by custom chemical solid phase peptide synthesis while adding a cysteine at the C-terminal, and conjugating a 1,4,7,10-tetraazacyclododecane-1,4,7,10-tetraacetic acid (DOTA) chelator via maleimide chemistry (Almac). The purified construct DOTA-Z_0185_ demonstrated to have > 97% purity by reverse-phase high-performance liquid chromatography (RP-HPLC) (Supplementary Figure S1A) and identity (7,106 g/mol) was confirmed by mass spectrometry (MS) (Supplementary Figure S1B). The precursor was vialled into 100 μg freeze-dried aliquots for further use and radiolabeling.

### Gallium-68 radiolabeling of DOTA-Z_0185_

**Figure 1:**
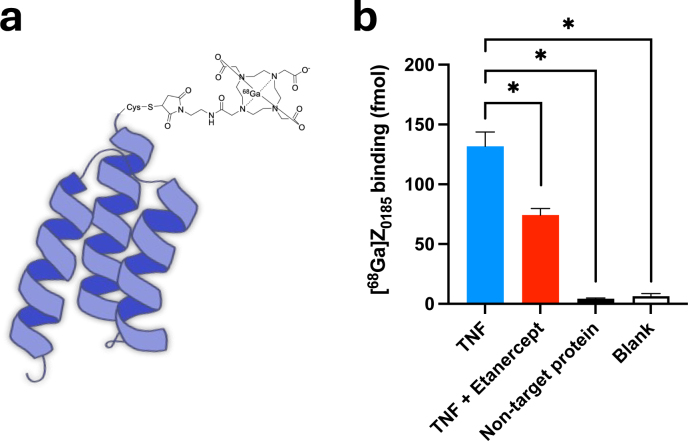
(a) Schematic structure of the PET tracer [^68^Ga]Ga-DOTA-Cys-Z_0185_ (here abbreviated as [^68^Ga]Z_0185_). (b) Binding of [^68^Ga]Z_0185_ to human TNF, either alone or after pre-blocking with etanercept. Binding toward a non-target protein and a blank is included as negative controls. ****P < 0.0001.

A ^68^Ge/^68^Ga generator was pre-eluted with hydrochloric acid (HCl), and the fraction with the highest radioactivity was used for radiolabeling. Gallium-68 eluate was mixed with 30 μg of DOTA-conjugated Z_0185_ in 2 M HEPES buffer (pH 3.3) and incubated at 65 °C for 5 min to allow for complex formation. The reaction mixture was purified using a NAP-5 size-exclusion column (Cytiva) with phosphate-buffered saline (PBS) containing 10% ethanol as the eluent. The final product, [^68^Ga]Z_0185_ (Figure 1a) was consistently obtained with a radiochemical purity >95%, as determined by HPLC (Supplementary Figure S2A, B). The molar activity of [^68^Ga]Z_0185_ was normally around 10 MBq/nmol at end of synthesis.

### TNF radioimmunoassay

A radioimmunoassay was developed to verify binding of [^68^Ga]Z_0185_ to recombinant TNF. Copper Coated High-Capacity Plate wells were coated with 10 μg/ml his-tagged TNF by incubating for 1 h at room temperature, then washed three times with 200 μL PBS, 0.05% Tween^®^-20 Detergent. As a negative control, other wells were coated with a structurally unrelated protein (his-tagged DGCR2) to which Z_0185_ was not expected to bind. Preblocking of non-specific sites was achieved by incubating wells with 100 μL PBS + 1% BSA for 15 min followed washing. Empty well without protein was used as blank to determine background binding. The fusion protein Etanercept (100 nM) was added to wells covered with TNF to act as blocker. [^68^Ga]Z_1085_ (corresponding 1 nmol) was then added to each well and allowed to incubate for 60 min at room temperature. Each condition was performed in eight individual replicates. Each well was washed three times with PBS, and next the radioactivity was measured in a well counter (inhouse build, Uppsala PET Center).

### Animal housing and general care

Male Sprague Dawley rats (Taconic; RRID: RGD_1566440) (200–250 g) were housed in pairs per cage with GLP Aspen Bedding (TAPVEI). Animals had ad libitum access to standard chow and water. Housing conditions were maintained at 22 °C and 50% humidity under a 12-h light/dark cycle. All animal procedures were approved by the Animal Ethics Committee of the Swedish Animal Welfare Agency (approval number: 5.8.18-09018/2020). All experiments were conducted in compliance with the ARRIVE guidelines (Animal Research: Reporting of In Vivo Experiments) and Uppsala University’s institutional policies on animal experimentation (UFV 2007/724).

### Bleomycin rat model of lung injury

Lung inflammation was induced via a single intratracheal instillation of BLM (1,500 IU in 200 μL saline; 2 mg/kg) under anesthesia by isoflurane inhalation in 12 male Sprague Dawley rats. Animals were monitored daily for 4 days following administration. An additional control group consisting of 10 healthy male Sprague Dawley rats was included for comparative analyses.

#### Ex vivo biodistribution

All rats received an intravenous injection of approximately 5 MBq [^68^Ga]Z_0185_ and were euthanized 1 h post-injection. Major organs, including the lungs, muscle, and spleen were excised, weighed, and their radioactivity measured using a gamma counter. After radioactivity measurement, lungs were fixed in formaldehyde and further processed for immunohistochemical (IHC) analysis.

#### Ex vivo autoradiography

Lungs were collected from rats injected with [^68^Ga]Z_0185_, after measurement in well counter. Tissues were snap-frozen, embedded in optimal cutting temperature (OCT) compound, and cryosectioned at 20 μm using a Micron HM560 cryostat (Germany). Tissue sections were mounted on Superfrost Plus slides (Menzel-Gläser). The entire process took approximately 1 h. The slides were then exposed to a phosphor-imaging plate (BAS-MS, FujiFilm) overnight and scanned using a phosphor imager (Amersham Typhoon FLA 9500, GE). Images were visualized with ImageJ (NIH, US).

#### PET/CT imaging of bleomycin treated and control rats

Post-mortem PET/computed tomography (CT) scans were performed on four BLM-treated and six control rats. One hour after tracer injection (5 MBq [^68^Ga]Z_0185_), rats were euthanized and a 40-min static PET/CT scan was conducted to evaluate tracer distribution in lung tissue. After the PET scan, tissues were collected, and measured for radioactivity by well counter as described above.

#### PET/CT analysis

PET/CT images were analyzed using PMOD 4.0 software (PMOD Technologies). Tissue segmentation was performed on fused PET/CT images and SUV_mean_ values were extracted from the PET images. The density of tissue from each segmentation was extracted from the CT images. The tissue fraction *k* was calculated from some lung segmentations using the formula *k* = (HU_Lesion_ – HU_Air_) / (HU_Tissue_ – HU_Air_) (9). The PET signal quantified as SUVmean could then by corrected for tissue density using the formula SUV_corr_ = SUV_mean_/*k*.

#### Immunohistochemistry and staining

Rat tissue biopsies were fixed in 4% paraformaldehyde for 24 h, followed by dehydration in 70% ethanol and paraffin embedding. Paraffin sections (4 μm thick) were stained with hematoxylin and eosin (H/E) and SIR at Uppsala University Hospital using standard protocols. IHC staining for CD68 was performed using the Autostainer Link 48 system and the EnVision FLEX High pH detection kit (Agilent). Antigen retrieval was carried out using the PT-Link system (Dako) with a high-pH (pH 9) Target Retrieval Buffer. Tissue sections were incubated with a recombinant anti-CD68 antibody (RRID: AB_323706, 1:100 dilution) for 60 min, followed by detection using a horseradish peroxidase-conjugated secondary antibody. Slides were scanned at 20× magnification using a NanoZoomer S60 digital slide scanner (Hamamatsu), and image analysis was performed using QuPath software (version 0.2.3).

### Statistical methods

Data are expressed as mean ± standard deviation (SD), with ‘n’ indicating the number of animals per group. Normality was tested using the Shapiro–Wilk test, and variances equality was assessed with an F-test. A *P*-value < 0.05 was considered statistically significant (*P < 0.05, **P ≤ 0.01, ***P ≤ 0.001). All statistical analyses were performed using GraphPad Prism version 10.1 (GraphPad Software, San Diego, CA, USA). Detailed statistical information is provided in the supplementary material (Supplementary Tables S1–S4).

## Results

### TNF radioimmunoassay

[^68^Ga]Z_0185_ bound to human recombinant TNF, in a manner that could be partially blocked by TNF-inhibitor etanercept (131.8 ± 12.0 fmol for [^68^Ga]Z_0185_ alone vs. 74.2 ± 5.6 fmol for etanercept co-incubation, *P* < 0.05) (Figure 1b). Binding of [^68^Ga]Z_0185_ to negative controls, including a protein unrelated to TNF, was low (4.2 ± 0.7 fmol), indicating minimal non-specific binding.

#### BLM model validation

**Figure 2:**
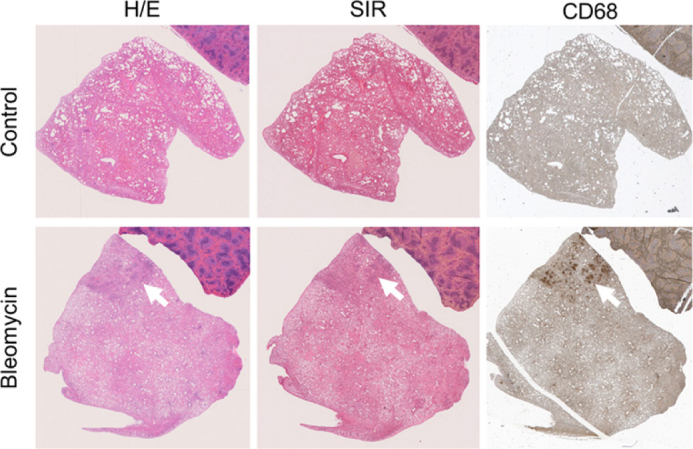
Representative histological analysis of lung tissue from control and bleomycin-treated rats. Sections were stained with hematoxylin and eosin (H/E) to assess tissue architecture and injury, Sirius Red (SIR) to visualize collagen deposition, and CD68 immunohistochemistry (CD68) to detect macrophage infiltration.

Lung tissue from BLM-treated rats exhibited distinct lesions, characterized by dense regions within the parenchyma. These areas showed marked collagen accumulation, as demonstrated by Sirius Red (SIR) staining, and co-localized with macrophage infiltration identified by cluster of differentiation 68 (CD68) immunohistochemistry (Figure 2). By contrast, lungs from healthy animals displayed normal structure, limited deposition of collagen and minimal CD68 positivity (Figure 2).

#### [^68^Ga]Z_0185_ uptake in lung of bleomycin treated rats

**Figure 3:**
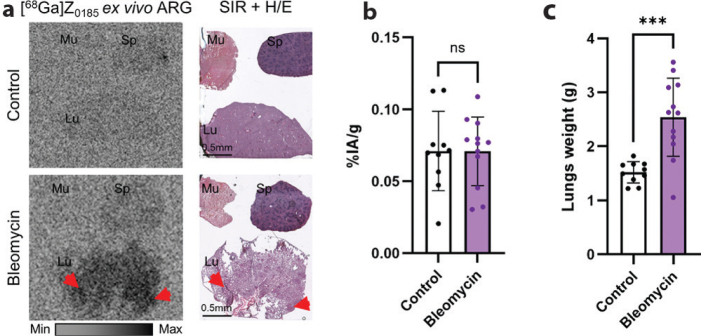
Ex vivo uptake of [^68^Ga]Z_0185_ in the bleomycin-treated rats. (a) Ex vivo ARG of lung sections from control and BLM rats, 1 h post-injection of the tracer. The red arrows indicate the tracer uptake in lungs’ lesions. (b) Ex vivo biodistribution data showing tracer uptake in the lungs of control and BLM rats, expressed as percentage of injected activity per gram of tissue (%IA/g). (c) Lung weights from control and BLM rats. ***P = 0.0006 IA, injected activity; Lu, lung; Mu, muscle; Sp, spleen.

To investigate the in vivo uptake of [^68^Ga]Z_0185_ in inflamed lung tissue, a single intratracheal dose of BLM was administered to induce lung injury in rats. Four days post-administration, both BLM-treated and control rats received an intravenous injection of approximately 5 MBq of [^68^Ga]Z_0185_ and were euthanized 1 h later for ex vivo and imaging analyses (Figure 3).

At the time of tissue collection, lungs from BLM-treated rats appeared enlarged, suggesting the presence of edema and inflammation. Quantitative analysis confirmed a significant increase in lung weight in the BLM group compared to controls (2.54 ± 0.73 g vs. 1.52 ± 0.20 g, *P* = 0.0006) (Figure 3c). Ex vivo autoradiography (ARG) performed on BLM lungs sections showed localized accumulation of [^68^Ga]Z_0185_ in pulmonary lesions (Figure 3a). However, ex vivo biodistribution, which quantify only the whole-lung tracer uptake, revealed no significant difference between control and BLM-treated animals (0.07 ± 0.03 IA%/g vs. 0.07 ± 0.02 IA%/g, respectively) (Figure 3b).

**Figure 4:**
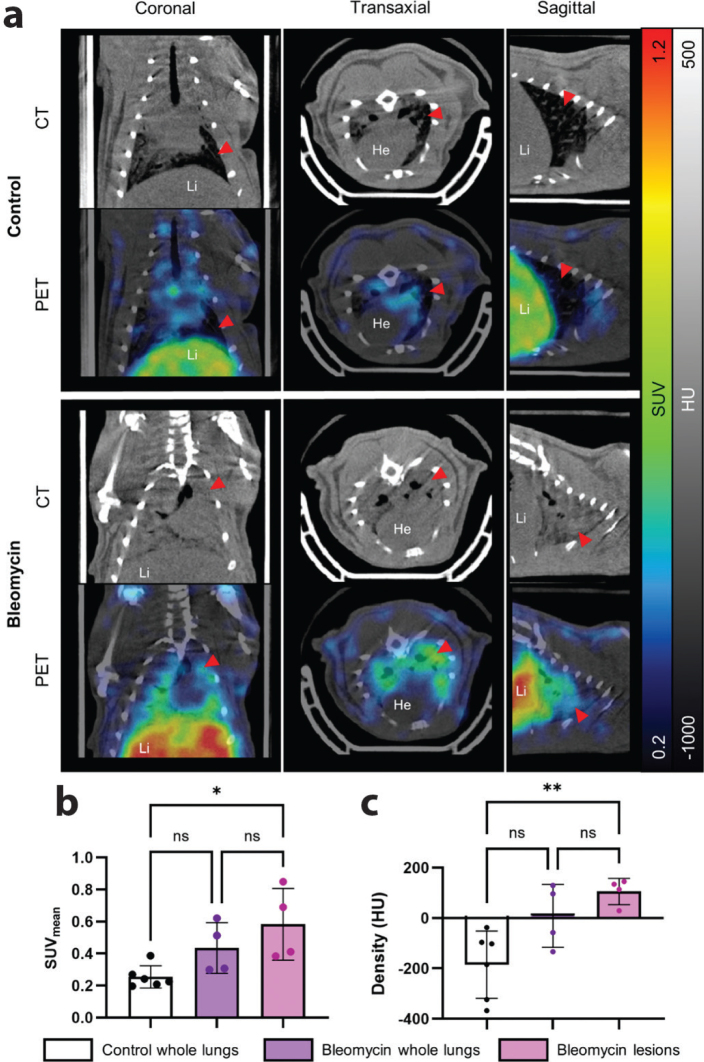
In vivo uptake of [^68^Ga]Z_0185_ in bleomycin-treated rats. (a) Representative PET images showing lung uptake of the tracer in control and BLM rats. The red arrows indicate the lungs. (b) Quantification of tracer uptake (SUV_mean_) from PET images, and (c) CT-derived tissue density measurements in whole lungs of control and BLM rats, as well as in lesions from BLM rats. *P < 0.05 and **< 0.01. He, heart; Li, liver.

To further evaluate regional uptake and tissue characteristics, post-mortem PET/CT imaging was performed (Figure 4). Whole-lung CT density measurements revealed no statistically significant differences between control and BLM groups. However, analysis of distinct pulmonary lesions within the BLM lungs showed significantly elevated Hounsfield Units (HU) compared to healthy lung regions (105.5 ± 52.3 HU vs. –185.3 ± 133.4 HU, *P* = 0.006) (Figure 4c). Moreover, [^68^Ga]Z_0185_ uptake co-localized with regions of increased CT density (Figure 4a). Quantification of PET signal revealed that SUV_mean_ in lesions was significantly higher than in control lungs (0.58 ± 0.22 vs. 0.25 ± 0.07, *P* = 0.016) (Figure 4b). In contrast, the mean standardized uptake value (SUV_mean_) for the whole BLM lung showed only a modest, non-significant increase compared to controls (0.43 ± 0.16).

Change in density of the lung may affect the quantification of the PET signal. After correction for tissue density, the signal SUV_corr_ in lesions remained almost twice as high as control lung (0.56 ± 0.23 vs. 0.34 ± 0.14). However, difference was not significant. There was no difference in [^68^Ga]Z_0185_ PET uptake or density in circulation or other major organs and tissues (Supplementary Figure 3A, B).

## Discussion

In this study, we demonstrate that the Affibody molecule-based PET tracer [^68^Ga]Z_0185_ enables specific detection of TNF expression in inflamed lung tissue using the BLM-induced rat model of pulmonary injury. While whole-lung uptake of [^68^Ga]Z_0185_ was similar between BLM-treated and control animals, we observed significantly higher focal tracer accumulation in injured regions. This suggests that [^68^Ga]Z_0185_ could serve as a non-invasive tool for detecting localized TNF-driven inflammation.

The BLM rat model exhibits an acute inflammatory phase within the first 7–10 days post-instillation, followed by fibrotic remodeling (10, 11). Previous studies have shown that TNF expression increases within this early phase, with elevated TNF levels in bronchoalveolar lavage fluid and increased release from alveolar macrophages around day 4 (12, 13). Our results are consistent with these findings. At day 4 post-instillation, BLM-treated rats showed significantly increased lung weight, suggestive of pulmonary edema. Histological and CT analyses revealed localized areas of increased tissue density, corresponding to lung injury. These areas exhibited CD68+ macrophage accumulation, confirming localized immune cell infiltration. Autoradiography further demonstrated specific [^68^Ga]Z_0185_ binding in these injured regions, indicating localized elevated TNF levels in inflamed tissue. Finally, PET imaging revealed lesion-specific uptake of [^68^Ga]Z_0185_ in BLM lungs. However, whole-lung SUV_mean_ values did not differ significantly between BLM and controls, underscoring the importance of lesion-level analysis in heterogeneous diseases like lung injury, where signals from localized inflammation may be masked by surrounding healthy tissue. After correction of the PET quantification using the tissue fraction there remained an almost twice as high signal in BLM lesions and healthy control lung. However, it was not significant as the correction introduced larger variability in all groups, especially in the healthy controls. Further optimization such as using a stand-alone preclinical μCT system with higher resolution and improved quantification may be used in future studies.

TNF plays a pivotal role in several inflammatory lung diseases, including IPF, chronic obstructive pulmonary disease, sarcoidosis, and acute lung injury (1). In IPF, TNF levels in the lungs have been correlated with fibrosis (14), and preclinical studies support a pro-fibrogenic role for TNF (15, 16). However, clinical trials evaluating TNF inhibitors in IPF patients, such as etanercept, failed to demonstrate significant improvement in lung function, although a non-significant reduction in disease progression was observed (17). These inconclusive clinical results may in part reflect the lack of sensitive methods to evaluate fibrogenesis, which limits assessment of treatment efficacy. Interestingly, in contrast to its pro-fibrotic role, TNF administration in mice accelerates the resolution of established pulmonary fibrosis in mice (18). This duality highlights the complex, context-dependent role of TNF in pulmonary disease.

In sarcoidosis, TNF is a well-established driver of inflammation, with alveolar macrophages releasing TNF that contributes to granuloma formation (19). Anti-TNF therapies such as infliximab, adalimumab and golimumab have demonstrated efficacy in many refractory cases, improving both symptoms and lung function (2–5). However, treatment responses vary, and some clinical trials have not demonstrated consistent benefit (20, 21).

Importantly, imaging studies using a TNF-targeting radiotracer, such as ⁹⁹ᵐTc-infliximab, have shown selective uptake in lung lesions of sarcoidosis patients, correlating with clinical parameters (22). In contrast, other patients with high [^18^F]FDG uptake exhibited no infliximab signal, suggesting that not all inflammation is TNF-mediated (23). These findings highlight the clinical value of TNF-targeted imaging for identifying patients most likely to benefit from anti-TNF therapy, and in the monitoring of treatment efficacy in clinical trials.

While [^18^F]FDG PET is commonly used to assess inflammation, it measures general metabolic activity and cannot distinguish between different inflammatory pathways. In contrast, TNF-targeted tracers offer pathway-specific insight.

Previous studies have investigated TNF-targeting radiotracers, primarily using large protein-based tracers such as monoclonal antibodies (infliximab, ~150 kDa) (22–25), Fc-fusion proteins (etanercept, ~150 kDa) (26), or PEGylated Fab fragments (certolizumab pegol, ~90 kDa) (27). Although these agents exhibit high affinity for TNF, their large size limits tissue penetration and results in slow pharmacokinetics. A smaller anti-TNF VHH-based single-photon emission CT (SPECT) tracer has been described, yet also displayed delayed imaging kinetics (28).

In contrast, the [^68^Ga]Z_0185_ tracer used in this study is based on a small Affibody molecule (7.1 kDa) with subnanomolar affinity for human TNF (7). Its low molecular weight allows for a rapid tissue penetration and clearance, enabling early imaging and labeling compatibility with short-lived radionuclides such as gallium-68. These properties may overcome limitations of previous tracers and enable more practical application in both preclinical and clinical settings.

In this study, we employed a widely used and well-characterized model of lung inflammation.

Bleomycin is the most widely used and best-characterized animal model for IPF due to its ability to replicate many pathological aspects of the human disease. The first week following bleomycin administration is characterized by acute injury, immune cell infiltration and pro-inflammatory cytokine release including TNF. However, there are also translational limitations of the bleomycin model, such as a stronger inflammatory component in rodents compared to IPF. Additionally, unlike the progressive nature of human IPF, fibrosis in mice may resolve spontaneously with time. Especially single-dose models may not capture the long-term chronic decay as seen in human IPF.

Here, we observed some inter-individual variability in lung injury severity among BLM-treated animals, as evidenced by histological evaluation (Supplementary Figure S4). This heterogeneity may have led to an underestimation of the tracer’s efficacy due to uneven TNF expression across individuals. Furthermore, only male rats were included in the study, limiting our ability to assess potential sex-related influences on the results. Despite these limitations, this study provides a valid proof of concept and demonstrates the potential of [^68^Ga]Z_0185_ for non-invasively assessing TNF-driven inflammation in the lungs.

A potential drawback with the current tracer construct, in the context of further development toward the clinic, is the reliance on Gallium-68 for radiolabeling. Although common in the clinic for labeling of peptides, Gallium-68 has relatively short half-life (68 min) and high positron energy resulting in suboptimal spatial resolution. We have previously presented [^18^F]AlF-RESCA-Z_0185_, a Fluorine-18 labeled version of the same Affibody molecule evaluated here (29). Fluorine-18, which has longer radioactive half-life (110 min) and provides excellent spatial resolution, may be an option to Gallium-68 for further development for imaging of TNF.

## Conclusions

We have demonstrated that the PET tracer [^68^Ga]Z_0185_, which targets TNF, accumulated in inflamed regions of lung tissue in BLM-treated rats, with uptake correlating to histological and CT features of injury. These findings support the potential of [^68^Ga]Z_0185_ as a non-invasive imaging tool for visualizing TNF-driven inflammation in the lungs. Such a tracer could improve disease diagnosis, patient stratification for anti-TNF therapies, and serve as a valuable biomarker in clinical trials for monitoring therapeutic response.

## Supplementary Material



## Declarations

### Availability of data and materials

The datasets used and/or analyzed during this study are available from the corresponding author on reasonable request.
