# Supplementary material for: Positron Emission Tomography imaging of tumor necrosis factor in lung injury
Source: Ups J Med Sci. 2026 Apr 28;131:10.48101/ujms.v131.13817. doi: 10.48101/ujms.v131.13817 (PMC13142652; doi:10.48101/ujms.v131.13817)
Supplement: Supplementary file 1 [file UJMS-131-13817-s1.pdf]

Supplementary Data

Positron Emission Tomography imaging of tumor necrosis factor in lung injury

Olivia Wegrzyniak et al.

Supplementary Figures and Tables

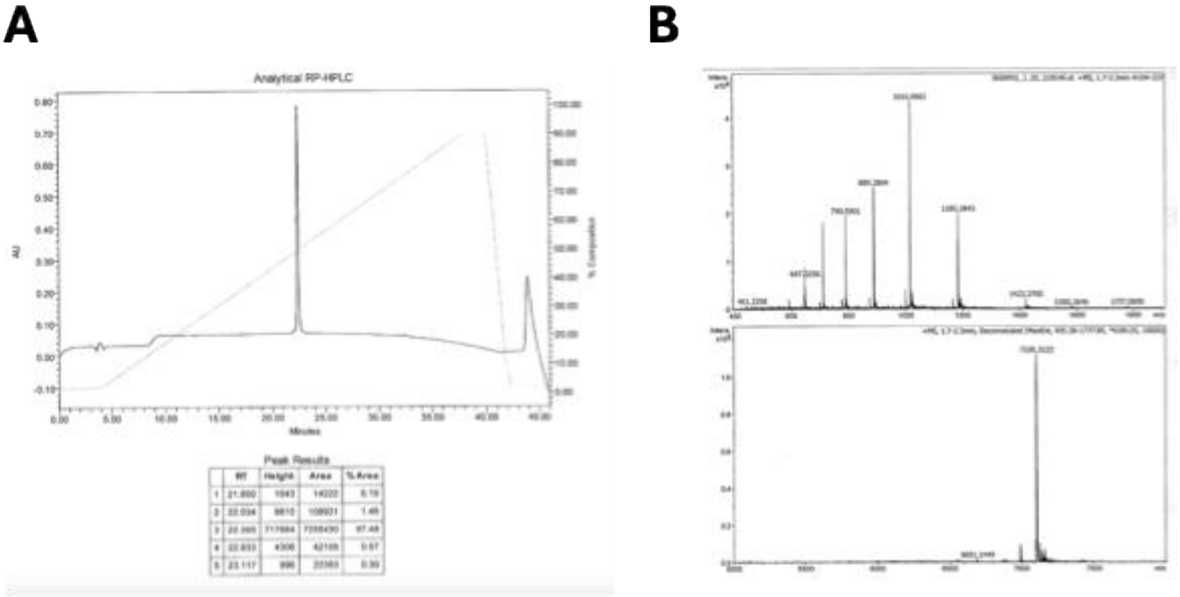

Supplementary Figure S1. (A) Purity of DOTA-Z_{0185} peptide precursor assessed by RP-HPLC. (B) Identity and molecular weight (7106 g/mol) confirmed by Mass Spectrometry (MS).

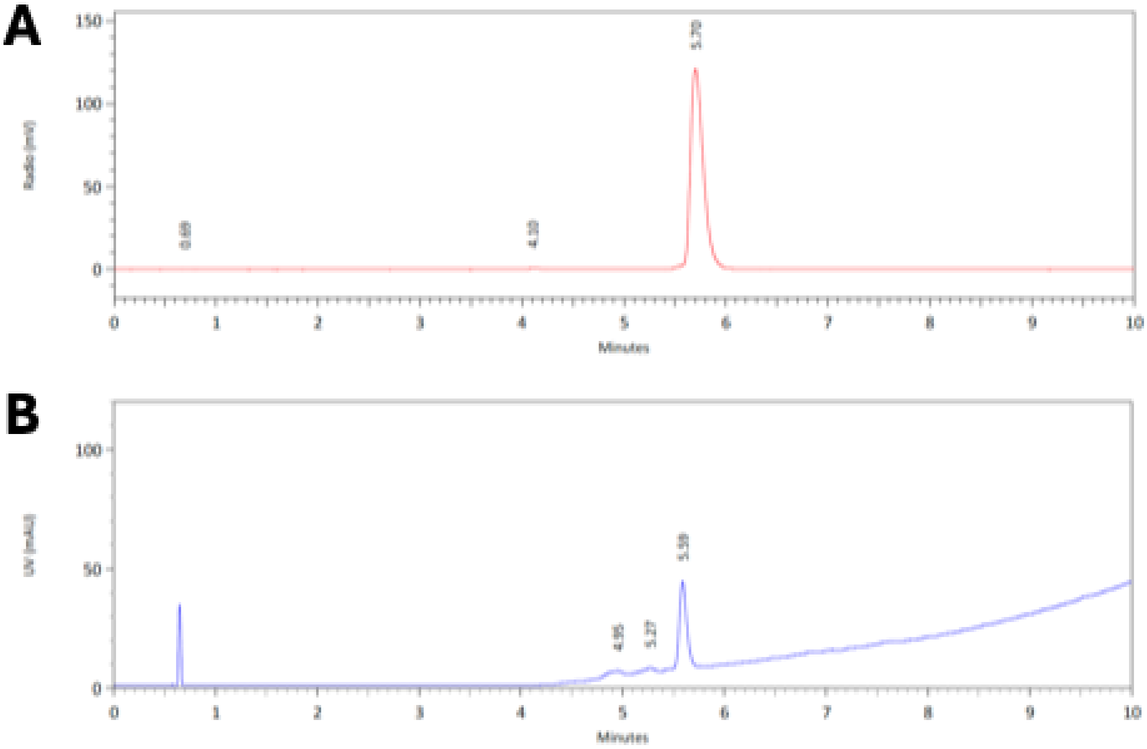

Supplementary Figure S2. Quality control of Gallium-68 radiolabeling of DOTA-Z_{0185} include (A) radio-chromatogram demonstrating excellent radiochemical purity and (B) similar retention time of the precursor peptide on UV (≈5.6 min).

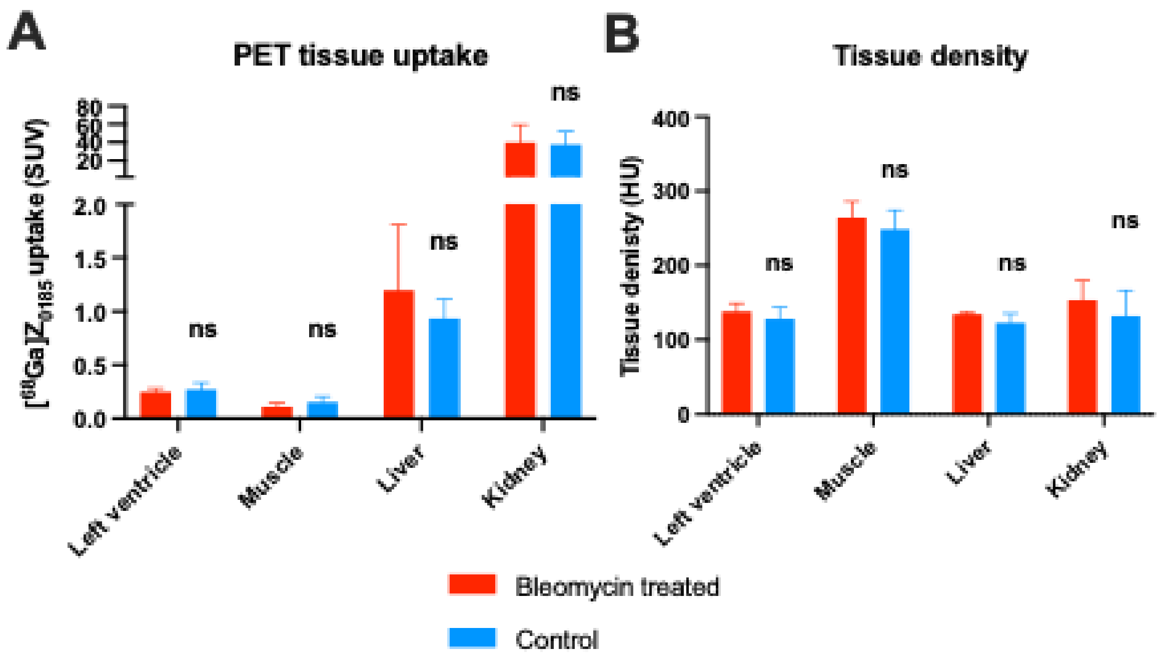

Supplementary Figure S3. Uptake of [^{68}Ga]Z$_{0185}$ in a selection of other major organs assessed by PET, showing no difference between bleomycin treated animals and healthy control animals (A). Tissue density in other major organs and tissues assessed by CT (B).

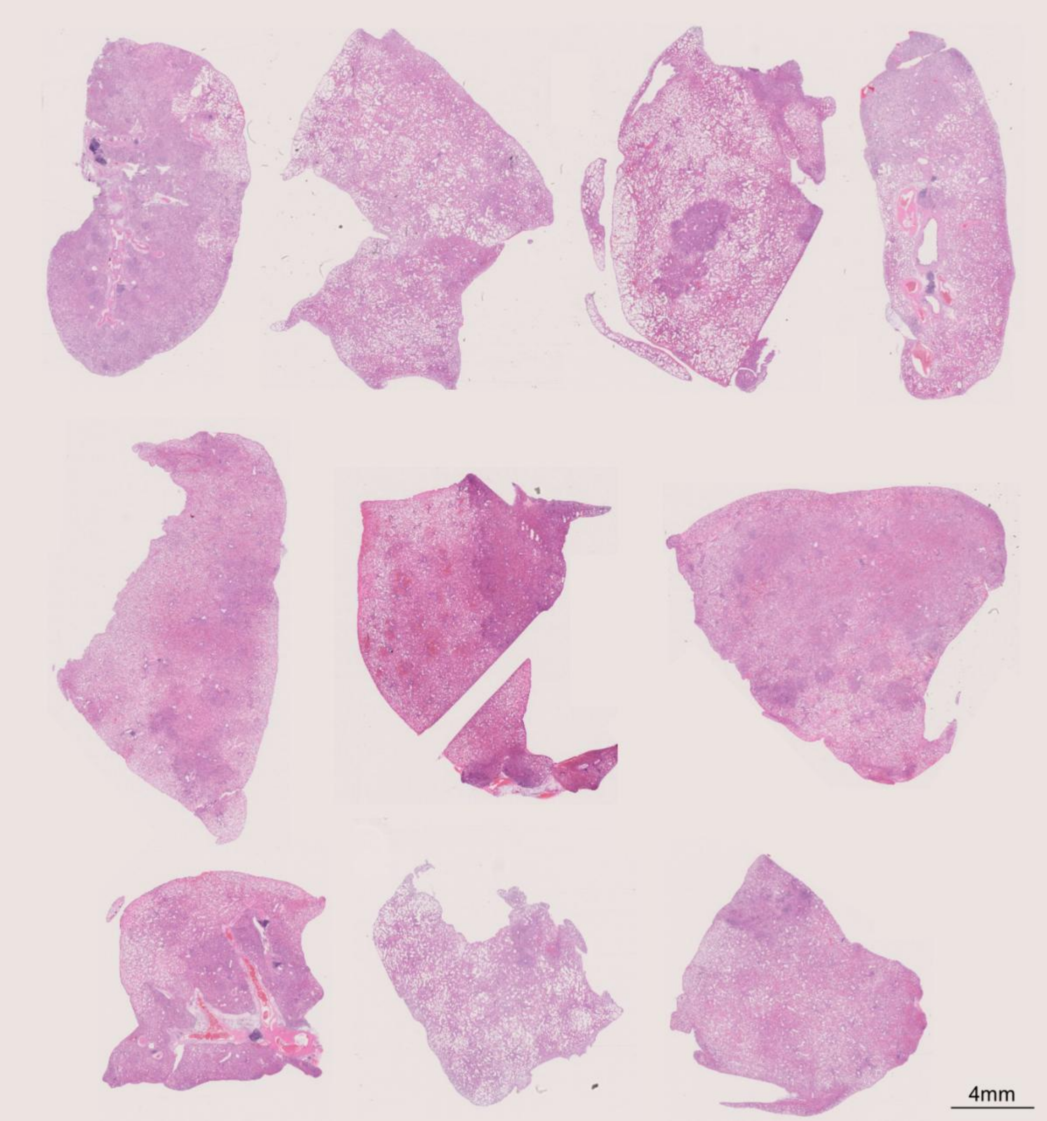

Supplementary Figure S4. Hematoxylin and eosin (HE) staining of lung tissue sections from *n* = 10 rats, 4 days after BLM instillation (40× magnification). The images illustrate inter-individual variability in lung injury severity among BLM-treated animals included in this study.

Table S1. Unpaired t-test comparing whole **lungs tracer uptake extracted from the ex vivo biodistribution** between **control (n=10) and BLM rats (n=12)** (related to **Figure 3B**).

	Weight lungs Control	Weight lungs Bleomycin
**N**	**10**	**12**
**mean**	0.071	0.071
**P value**	**0.984**	
**t, df**	**t=0.02, df=20**	
**95% CI**	-0.023 to 0.023	

Table S2. Unpaired t-test with Welch's correction comparing **weight of lungs** between **control (n=10) and BLM rats (n=12)** (related to **Figure 3C**).

	Weight lungs Control	Weight lungs Bleomycin
**N**	**10**	**12**
**mean**	1.518	2.539
**P value**	**0.0006**	
**t, df**	**t=4.67, df=12.95**	
**95% CI**	0.548 to 1.494	

Table S3. Post-hoc **Tukey's multiple comparison test** comparing PET derived SUV_{mean} from the tracer in lungs from **healthy (n=6) and BLM rats (n=4)** (related to **Figure 4B**).

Tukey's multiple comparisons test	Mean 1	Mean 2	n1	n2	q	DF	95% CI of diff.	Adjusted P value
Control whole lungs vs. BML whole lungs	0.25	0.43	6	4	2.61	11	-0.44 to 0.08	0.200
Control whole lungs vs. BLM Lesions	0.25	0.58	6	4	4.77	11	-0.59 to -0.07	0.016
Bleomycin whole lungs vs. BLM Lesions	0.43	0.58	4	4	1.97	11	-0.43 to 0.14	0.379

Table S4. Post-hoc **Tukey's multiple comparison test** comparing tissue density from lungs from **healthy (n=6) and BLM rats (n=4)** (related to **Figure 4C**).

Tukey's multiple comparisons test	Mean 1	Mean 2	n1	n2	q	DF	95% CI of diff.	Adjusted P value
Control whole lungs vs. BML whole lungs	-185.3	8.8	6	4	3.72	11	-393.5 to 5.334	0.057
Control whole lungs vs. BLM Lesions	-185.3	105.5	6	4	5.57	11	-490.2 to -91.40	0.006
BLM whole lungs vs. BLM Lesions	8.749	105.5	4	4	1.69	11	-315.2 to 121.7	0.480
